# Alternative Culture Systems for Bovine Oocyte In Vitro Maturation: Liquid Marbles and Differentially Shaped 96-Well Plates

**DOI:** 10.3390/ani13101635

**Published:** 2023-05-14

**Authors:** Andrea Fernández-Montoro, Daniel Angel-Velez, Camilla Benedetti, Nima Azari-Dolatabad, Osvaldo Bogado Pascottini, Ann Van Soom, Krishna Chaitanya Pavani

**Affiliations:** 1 Department of Internal Medicine, Reproduction and Population Medicine, Ghent University, 9820 Merelbeke, Belgium; andrea.fernandezmontoro@ugent.be (A.F.-M.); daniel.angelvelez@ugent.be (D.A.-V.); camilla.benedetti@ugent.be (C.B.); nima.azaridolatabad@ugent.be (N.A.-D.); osvaldo.bogado@ugent.be (O.B.P.); krishnachaitanya.pavani@ugent.be (K.C.P.); 2 Research Group in Animal Sciences—INCA-CES, Universidad CES, Medellin 050021, Colombia; 3 Department for Reproductive Medicine, Ghent University Hospital, 9000 Gent, Belgium

**Keywords:** oocyte, in vitro maturation, culture systems, assisted reproduction technologies, bovine

## Abstract

**Simple Summary:**

Oocyte maturation is a complex process in which the oocyte acquires the capacity to be fertilized and to support embryo development. Although oocyte maturation in vitro has undergone significant advancements, the systems that are currently in place still need to be further optimized. This study aimed to examine alternative culture systems for bovine oocyte in vitro maturation. Specifically, we used liquid marbles and differentially shaped 96-well plates (flat, v-shaped, and round-bottom). Both systems were able to support oocyte nuclear maturation, but embryo development was reduced when in vitro maturation was performed in liquid marbles. Differently shaped wells during maturation did not affect embryo yield but presented a reduction in blastocyst quality. Hence, 96-well plates might be an alternative culture system to mature oocytes in small groups, but further analyses to test possible toxic effects are needed.

**Abstract:**

In vivo-matured oocytes exhibit higher developmental competence than those matured in vitro but mimicking the in vivo environment by in vitro conditions has been challenging. Until now, conventional two-dimensional (2D) systems have been used for in vitro maturation of bovine cumulus-oocytes-complexes (COCs). However, using such systems present certain limitations. Therefore, alternative low-cost methodologies may help to optimize oocyte in vitro maturation. Here, we used two different systems to culture COCs and evaluate their potential influence on embryo development and quality. In the first system, we used treated fumed silica particles to create a 3D microenvironment (liquid marbles; LM) to mature COCs. In the second system, we cultured COCs in 96-well plates with different dimensions (flat, ultra-low attachment round-bottom, and v-shaped 96-well plates). In both systems, the nuclear maturation rate remained similar to the control in 2D, showing that most oocytes reached metaphase II. However, the subsequent blastocyst rate remained lower in the liquid marble system compared with the 96-well plates and control 2D systems. Interestingly, a lower total cell number was found in the resulting embryos from both systems (LM and 96-well plates) compared with the control. In conclusion, oocytes matured in liquid marbles or 96-well plates showed no remarkable change in terms of meiotic resumption. None of the surface geometries influenced embryo development while oocyte maturation in liquid marbles led to reduced embryo development. These findings show that different geometry during maturation did not have a large impact on oocyte and embryo development. Lower embryo production after in vitro maturation in liquid marbles was probably detected because in vitro maturation was performed in serum-free medium, which makes oocytes more sensitive to possible toxic effects from the environment.

## 1. Introduction

Although extensive research has been conducted in the last decades to improve the conditions for in vitro maturation (IVM) of bovine oocytes, the developmental competence of in vitro-matured oocytes is still suboptimal when compared with in vivo-matured oocytes [1,2]. One of the reasons why in vitro maturation is not always successful is that this process is extremely sensitive to environmental factors such as pH [3], temperature variations [4], oxygen tension [5], media composition [6], or type of culture (group or individual) [7]. If these factors are kept under control, another major difference is the lack of continuous nutrient supply or dynamic removal of waste; this can be mimicked in vitro by microfluidics culture, but such systems are tedious to develop and less user-friendly [8,9,10]. A third factor which is different during routine in vitro maturation is that oocytes are not cultured in a follicular shape, while in vivo, the oocyte is maturing inside the preovulatory follicle. 

Traditional methods for in vitro maturation rely on two-dimensional (2D) culturing techniques using plastic culture plates, which have been used for decades to propagate monolayer cell cultures [11]. In such conventional 2D systems, cells are grown on flat, firm culture substrates, which are economic and relatively easy to handle. However, in such culture conditions, cells can adhere and spread freely in the horizontal plane but they have limited possibility for spreading in the vertical dimension [12]. Hence, the major drawback of 2D-culture systems is that they do not fully imitate the in vivo microenvironment where cells are grown in a complex three-dimensional (3D) matrix, which has an impact on cell–cell and cell–extracellular matrix interactions, and consequently on cell responses (differentiation, proliferation, apoptosis, gene, and protein expression) [13,14,15,16,17,18]. Furthermore, the cell morphology in 2D systems is different from that in the natural structures of tissues, which might affect their functionality, secretion of growth factors, organization of internal structures, and cell signaling [19,20].

There is growing evidence suggesting that 3D cell culture models reflect more precisely the actual microenvironment in which cells grow in native tissues [21]. These models allow cell adhesion in all three dimensions, whereas in the 2D system, it is restricted to the x-y plan. Likewise, 3D systems improve cell communication and soluble factors are more stable in those systems compared with 2D systems [11,22]. In terms of in vitro embryo production, studies on 3D systems were designed to support oocyte IVM in several species using alginate microbeads [23], alginate hydrogels [24], glass scaffolds [25], agarose matrix [26], or the hanging drop method [27]. Recently, so-called liquid marbles (LM) have also become interesting as 3D-shaped bioreactors. Liquid marbles are culture medium droplets encapsulated with hydrophobic particles which prevent direct contact between the liquid inside and the surrounding environment, thus reducing the risk of contamination, while the hydrophobic shell of the LM remains permeable for gases [28]. These properties make LM a promising alternative as 3D microbioreactors for cell culture. This system has been used for culturing microorganisms [29], embryoid bodies [30,31], or olfactory ensheathing cell spheroids [32]. The use of liquid marbles during ovine IVM showed slightly better blastocyst rates using polytetrafluoroethylene [33] and silica powder [34], while in porcine, fluorinated ethylene propylene liquid marbles were able to maintain the 3D organization of the COCs [24]. However, liquid marbles have not been validated yet in the bovine model.

Cellular responses are influenced by topographical surface features. For instance, human epithelial cells presented differences in orientation, migration, and morphology when culturing them on pillar or pit surfaces [35]. Similarly, concave and convex surfaces have been found to influence stem cells’ differentiation into osteoblasts [36], and a v-shaped surface has been related to changes in cell shape and mRNA expression in fibroblasts [37,38] and osteoblast-like cells [39]. However, all these studies used complex systems to recreate the different surface geometries. Thus, a simpler alternative could be to use 96-well plates with different shapes, which are available on the market and are easy to use and standardize. V-shaped 96-well plates have been applied in ovarian follicle culture, allowing growth and differentiation of bovine [40] and human follicles [41]. Similarly, round-bottomed, ultra-low attachment plates have been used in mouse follicle culture in order to prevent the attachment and flattening of the follicles to the surface of the plate, showing that non-attachment culture conditions have an impact on cumulus cells gene expression and oocyte developmental competence [42]. Besides, the effect of three differentially shaped 96-well plates have been tested during culture of several human cell lines (retinal epithelial, alveolar epithelial, and dermal fibroblastic) [43]; nevertheless, to our knowledge, no studies have been performed with these plates to evaluate the potential influence of different surface topographies during oocyte IVM on embryo development.

In the present work, we evaluated the potential effects of using two alternative culture systems during oocyte IVM: (1) encapsulation in liquid marble microbioreactors and (2) differently shaped culture substrates (flat, round, and v-shaped 96-well plates). 

## 2. Materials and Methods

### 2.1. Experimental Design

#### 2.1.1. Experiment 1: Evaluation of Liquid Marbles as 3-D Model for In Vitro Maturation

Liquid marbles were tested as microbioreactors for in vitro maturation of bovine oocytes. To do so, a total of 941 cumulus-oocyte complexes (COCs) in seven replicates were used in three different maturation systems: liquid marbles (LM) (*n* = 301 COCs) as the 3D model, 2D droplets (*n* = 309 COCs) as the flat culture with similar oocyte/medium ratio to LM (5 COCs/30 μL), and the control group (*n* = 331 COCs) as our standard condition (60 COCs/500 μL). After maturation, COCs from all groups were randomly distributed for in vitro fertilization (IVF) and in vitro culture (IVC; LM = 241, 2D droplets = 256, and Control = 262 COCs) or nuclear maturation assessment (LM = 60, 2D droplets = 53, and Control = 69 COCs).

#### 2.1.2. Experiment 2: Evaluation of Different Surface Geometries for In Vitro Maturation

A comparative study was carried out to evaluate the effect of three different bottom-shaped multi-well plates during oocyte IVM on embryo development. Firstly, we conducted a pilot study including 3 replicates (*n* = 1414 COCs) to select the ideal work volume of maturation medium per well and to analyze the effect of paraffin oil overlay in oocytes matured in multi-well plates. Cumulus-oocyte complexes were matured in v-shaped 96-well plates under the following conditions: five COCs in 30 μL maturation medium with 30 μL oil overlay (Oil V-shaped-30 (OV-30); *n* = 160 COCs) or without oil (V-shaped-30 (V-30); *n* = 120 COCs), ten COCs in 60 μL maturation medium with 30 μL oil overlay (OV-60; *n* = 163 COCs) or without oil (V-60; *n* = 155 COCs), and twenty COCs in 120 μL maturation medium with 30 μL oil overlay (OV-120; *n* = 143 COCs) or without oil (V-120; *n* = 148 COCs). A control group (*n* = 252 COCs) was also included. After maturation, IVF and IVC were performed routinely. 

**Figure 1:**
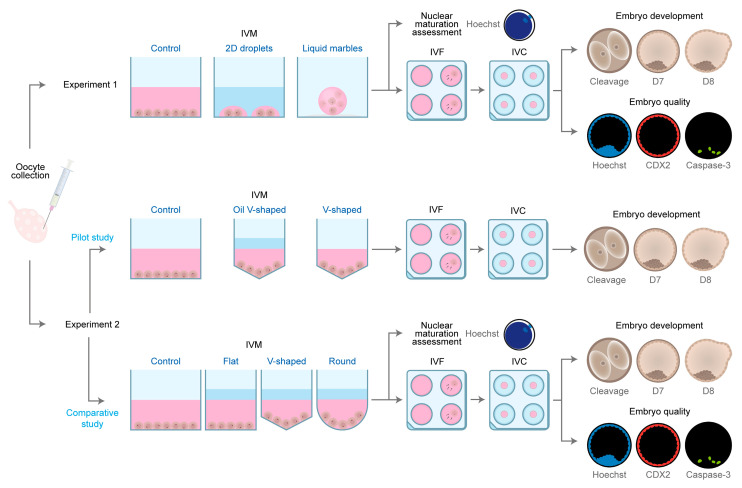
Schematic representation of the experimental design.

Subsequently, based on the results of the pilot study, different surface geometries were compared during maturation using three differentially shaped 96-well plates: flat (F), which can be represented as a cylindrical structure; ultra-low attachment round-bottom (R), with the appearance of a cylinder on the top and a semi-hemisphere on the bottom; and v-shaped (V); which can be represented as a cylinder on the top and a cone on the bottom. Besides evaluating the effect of closer interactions between COCs enhanced by the geometry of round and v-shaped 96-well plates (Figure S1), these plates also allow comparison of the impact of cell adhesion (i.e., the round-bottom plates present a covalently bonded hydrogel that minimizes cell attachment, while v-shaped plates are not coated). To do so, six replicates were performed (*n* = 1992 COCs) using the conditions described as for the OV-60, resulting in 4 groups: F-60 (*n* = 427 COCs), R-60 (*n* = 549 COCs), V-60 (*n* = 491 COCs), and control group (*n* = 525 COCs). After maturation, COCs from all groups were randomly assigned to IVF and IVC (F-60 = 374, R-60 = 504, V-60 = 440 and control group = 487 COCs) or nuclear maturation assessment (F-60 = 53, R-60 = 45, V-60 = 51 and control group = 38 COCs). The experimental design is depicted in Figure 1.

### 2.2. Media and Reagents

Tissue culture media (TCM)-199 and antibiotics (gentamycin and kanamycin) were obtained from Life Technologies Europe (Ghent, Belgium). Phosphate-Buffered Saline (PBS) was purchased from Gibco™ 20012019, Thermo Fisher Scientific (Waltham, MA, USA). All other products not indicated otherwise were provided by Sigma-Aldrich (Diegem, Belgium). Before use, every media was filtered (0.22 μM; GE Healthcare-Whatman, Diegem, Belgium).

### 2.3. Source of Oocytes and In Vitro Maturation

Bovine ovaries were obtained from a local slaughterhouse, transported to the laboratory, and prepared for further processing within 2 h after collection. The ovaries were disinfected with 96% ethanol and cleaned three times in physiological saline (37 °C) containing 50 mg/mL of kanamycin. Cumulus-oocyte complexes were recovered with an 18-gauge needle and a 10 mL syringe from 4–8-mm-diameter follicles. Oocytes surrounded by three or more layers of compact cumulus cells and a uniformly granulated cytoplasm were selected, washed in warm HEPES—Tyrode’s Albumin Lactate Pyruvate media (HEPES-TALP), and randomly assigned to different IVM systems. Four IVM systems, as described below, were evaluated according to the experimental group (see Section 2.1). All treatment groups were cultured for 22 h in 5% CO_2_ in the air at 38.5 °C.

Control. Sixty COCs were cultured in 500 μL maturation medium (TCM-199 Earle’s salts supplemented with 20 ng/mL epidermal growth factor and 50 μg/mL gentamicin) in flat-bottom 4-well dishes (Thermo Fisher^®^, Merelbeke, Belgium) without oil covering. 

**Figure 2:**
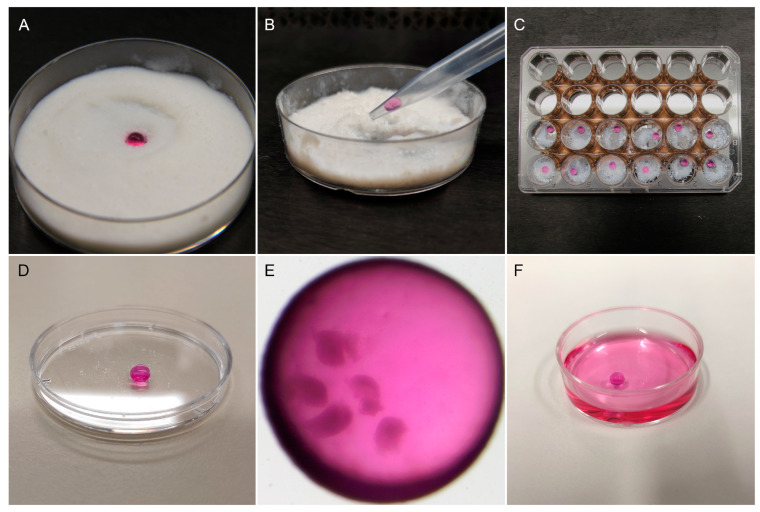
Oocyte encapsulation in liquid marbles. (**A**) One droplet of maturation medium containing the oocytes was placed in treated fumed silica powder on a Petri dish. The Petri dish was gently shaken to form the liquid marble. (**B**) A modified 1000 µL micropipette tip was used to manipulate the liquid marbles. (**C**) Liquid marbles were placed individually in the wells of a 24-well plate containing a small amount of silica powder. The central space of the plate was filled with 5 mL HEPES-TALP to prevent evaporation. (**D**) Resulting liquid marble drop. (**E**) Five COCs encapsulated in a liquid marble drop before IVM, observed under a stereomicroscope. (**F**) After maturation, liquid marbles were dissolved in maturation medium.

Encapsulation in liquid marbles. A single droplet of 30 μL maturation medium containing five COCs was carefully placed on top of a layer of approximately 1 cm treated fumed silica powder (Cabot Corp, Cab-O-Sil, TS-530), which was equally distributed in a 6 cm Petri dish (Figure 2A). The Petri dish was mildly shaken in circular motions to ensure that the surface of the droplet was completely and uniformly coated with the hydrophobic particles. To manipulate the LM, the edge of a 1000 µL micropipette tip was cut to make its diameter to a small extent of the LM diameter in order to ensure a proper grip, but big enough to avoid collapse. Before transferring the marbles, the modified tip was coated with some powder to prevent its adhesion to the tip. Then, the LM was picked up slowly (Figure 2B) and placed on a well of a 24-well plate (Thermo Scientific) whose surface was previously covered with a small quantity of silica powder (Figure 2C). To avoid evaporation, the central space of the 24-well plate was filled with 5 mL sterile HEPES-TALP medium. After IVM, the LM (Figure 2D,E) was placed in maturation medium to disrupt the silica powder’s hydrophobicity, causing the marble’s dissolution (Figure 2F). The released COCs were washed three times in maturation medium to remove silica particles before proceeding to the next step.

2D droplets. Droplets of 30 μL maturation medium were prepared in a Petri dish (60 × 15 mm; Thermo Fisher Scientific, Waltham, MA USA) and covered with 7.5 mL paraffin oil (SAGE, CooperSurgical, Trumbull, CT, USA). Five COCs were matured in each droplet of maturation medium.

Shaped culture in 96-well plates. Firstly, oocytes were matured in v-shaped 96-well plates under the following conditions: five COCs in 30 μL of maturation medium with or without paraffin oil overlay of 30 μL, ten COCs in 60 μL of maturation medium with or without a paraffin oil overlay of 30 μL, and twenty COCs in 120 μL of maturation medium with or without a paraffin oil overlay of 30 μL. Secondly, ten COCs were matured in 60 μL of maturation medium with paraffin oil overlay in flat, ultra-low attachment round-bottom, and v-shaped Corning^®^ 96-well plates (Avantor, VWR, Leuven, Belgium; CatNo. 734-1793, 444-1020, 734-1798, respectively).

### 2.4. In Vitro Fertilization and Embryo Culture

Standard in vitro methods were used to generate bovine embryos, as previously described by Wydooghe et al. [44]. Briefly, using a discontinuous 45/90% Percoll^®^ gradient (GE Healthcare Biosciences, Uppsala, Sweden), sperm capacitation of frozen-thawed straws from a known fertile bull was performed. Consequently, the sperm pellet was washed in IVF–TALP medium and a final concentration of 1 × 10^6^ spermatozoa/mL was adjusted using IVF–TALP medium enriched with BSA (Sigma A8806; 6 mg/mL) and heparin (20 μg/mL). 

After 22 h of IVM, oocytes from each treatment group in Experiments 1 and 2 were pooled to reach groups of 60 COCs. Then, oocytes were washed in IVF-TALP and subsequently co-incubated in 500 μL IVF-TALP with Percoll-purified spermatozoa for 21 h at 38.5 °C in 5% CO_2_ in humidified air. After fertilization, zona attached sperm and cumulus cells were removed by vortexing for 3 min in 2.5 mL Hepes-TALP. The presumed zygotes were randomly selected and cultured in groups of 25 in 50 μL droplets of synthetic oviductal fluid (SOF), 0.4% (*w*/*v*) BSA (Sigma A9647), and ITS (5 μg/mL insulin, 5 μg/mL transferrin, and 5 ng/mL selenium). Each droplet was covered with 900 μL paraffin oil and incubated at 38.5 °C for 8 days in 5% CO_2_, 5% O_2_, and 90% N_2_.

Cleavage was evaluated 45 h post insemination and blastocyst yield was recorded on day 7 and day 8 post insemination. Both rates were calculated as a percentage over the presumed zygotes. 

### 2.5. Evaluation of Oocyte Nuclear Stage (Maturation Assessment)

**Figure 3:**
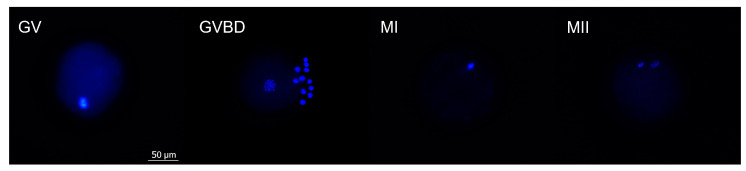
Representative images of Hoechst-stained oocytes in different nuclear maturation stages. (GV) Germinal vesicle. (GVBD) Germinal vesicle breakdown. (MI) Metaphase I. (MII) Metaphase II. The scale bar applies to all the images.

After maturation, oocytes were denuded by vortexing for 8 min in 2.5 mL Hepes-TALP and fixed with 4% paraformaldehyde (*w*/*v*). Then, oocytes were transferred to 0.1% (*w*/*v*) polyvinylpyrrolidone (PVP) in PBS containing 10 μg/mL of Hoechst 33,342 (Life Technologies, Ghent, Belgium) for 10 min. Nuclear morphology was evaluated using a fluorescence microscope (BRESSER Science ADL 601 F LED). The proportion of oocytes in each meiotic stage—germinal vesicle (GV), germinal vesicle breakdown (GVBD), metaphase I (MI), metaphase II (MII), or degenerated—was recorded (Figure 3). 

### 2.6. Embryo Quality Assessment

Embryo quality was determined by differential apoptotic staining for CDX2, a transcription factor only expressed by trophectoderm cells, and caspase-3, a cysteine-aspartic acid protease involved in the signaling pathways of cell apoptosis. The protocol was performed according to Wydooghe et al. [45]. Briefly, day-8 blastocysts were fixed in 4% paraformaldehyde (*w*/*v*) at room temperature for at least 20 min and then stored in PBS supplemented with 0.5% BSA at 4 °C until the staining was performed (Figures S2 and S3). Firstly, blastocysts were incubated with ready-to-use anti-CDX2 primary antibodies (Biogenex, San Ramon, CA, USA). Embryos were next incubated with rabbit active caspase-3 primary antibody (0.768 ng/mL, Cell Signaling Technology, Leiden, The Netherlands), followed by incubation in goat anti-mouse Texas Red secondary antibody (20 μg/mL in blocking solution, Molecular Probes, Merelbeke, Belgium) and then in goat antirabbit FITC secondary antibody (10 μg/mL in blocking solution, Molecular Probes). Finally, the embryos were transferred to nuclear stain, Hoechst 33,342 (50 μg/mL in PBS/BSA). A negative control was also included in which embryos were not incubated with CDX2 and active caspase-3 antibodies. Samples were examined by a single observer using fluorescence microscopy (Leica DM 5500 B) with a triple bandpass filter. With this staining protocol, the number of trophectoderm (TE) cells, inner cell mass number (ICM), total cell number (TCN = TE + ICM), ICM/TCN ratio, the total number of apoptotic cells (AC), and the ratio of apoptotic cells (ACR; AC/TCN) were estimated.

### 2.7. Statistical Analyses

The statistical analyses were performed using R-core (version 4.2.1; R Core Team, Vienna, Austria). The oocyte/zygote/embryo was considered as the unit of interest. Generalized mixed-effects models were used to test the effect of IVM conditions on oocyte nuclear maturation, cleavage, and embryo development rates. The effect of IVM conditions on blastocyst differential staining parameters was fitted in mixed linear regression models. For all the models, the replicate was set as random. Results are expressed as least square means and standard errors. The differences between treatment groups were assessed using Tukey’s post hoc test. The significance and tendency levels were set at *p* < 0.05 and *p* < 0.1, respectively.

## 3. Results

### 3.1. Experiment 1: Evaluation of Liquid Marbles as a 3D Model for In Vitro Maturation

#### 3.1.1. Effect of Liquid Marbles on Oocyte Nuclear Maturation

Nuclear maturation assessment by Hoechst staining demonstrated that oocytes in the three groups resumed meiosis (i.e., no germinal vesicles were found). Most oocytes in all treatment groups reached the metaphase II stage with no significant differences among groups (*p* > 0.05; Table 1). Likewise, the proportion of oocytes that reached germinal vesicle breakdown, metaphase I, or degenerated was similar among groups (*p* > 0.05).

#### 3.1.2. Effect of Liquid Marbles on Embryo Development and Embryo Quality

**Figure 4:**
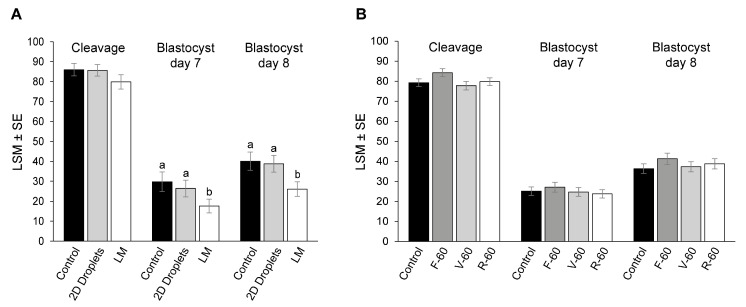
Cleavage, day 7, and day 8 blastocyst rates are expressed as a percentage of presumed zygotes. (**A**) Experiment 1. Oocytes were in vitro matured in liquid marbles (LM), 2D droplets, and a control group. (**B**) Experiment 2. Oocytes were in vitro matured in flat, v-shaped, and ultra-low attachment round-bottom 96-well plates, and a control group. Different superscripts (a and b) represent statistical differences (*p* < 0.05) among groups. Results are expressed as least square means ± standard error (LSM ± SE).

Firstly, we determine the effect of oocyte maturation conditions on cleavage and blastocyst rates. Although there were no significant differences in the cleavage rates between groups (LM: 79.9 ± 3.1%; 2D droplets: 85.7 ± 2.9%; control group: 86.0 ± 3.1%; *p* > 0.05; Figure 4A), oocytes matured in LM showed lower day 7 (17.6 ± 3.4%) and day 8 (26.1 ± 3.7%) blastocyst rates compared with 2D droplets (26.4 ± 4.2%, *p* = 0.048, and 38.8 ± 4.2%, *p* = 0.008, respectively) and control (29.8 ± 4.9%, *p* = 0.01, and 40.1 ± 4.6%, *p* = 0.007, respectively).

Further on, we determined the effect of maturation conditions on embryo quality by differential apoptotic staining of blastocysts. Differences among IVM culture systems in blastocyst quality parameters are shown in Table 2. Maturation in LM produced blastocysts with lower TCN and TE than in control (*p* < 0.01). Maturation in 2D droplets reduced the TCN, ICM, and TE (*p* < 0.01) and increased the AC/TCN ratio compared with control (*p* = 0.03).

### 3.2. Experiment 2: Evaluation of Different Surface Geometries for In Vitro Maturation

#### 3.2.1. Effect of Volume of Medium and Oil Overlay on 96-Well Plates Culture

Initially, a pilot study was performed to establish the optimum volume of maturation medium and the effect of paraffin oil overlay during oocyte IVM in 96-well plates on embryo development. The V-30 group was excluded from the treatment groups due to excessive maturation medium evaporation after IVM. Although there were no significant differences in the cleavage, blastocyst day 7, and blastocyst day 8 rates for all the treatment groups compared with the control (*p* > 0.05), OV-60 had a numerically higher blastocyst rate at day 8. Therefore, we selected IVM culture conditions as described for this group for follow-up experiments. For detailed information on the pilot study results, see Supporting Information (Table S1).

#### 3.2.2. Effect of Surface Geometry on Oocyte Nuclear Maturation, Embryo Development, and Embryo Quality

Hoechst staining was used to analyze the meiotic progression of the oocytes after IVM in flat, v-shaped, and ultra-low attachment round-bottom 96-well plates. No differences were found in the proportion of mature, immature, or degenerated oocytes in the three tested geometries and the control group (*p* > 0.05; Table 3).

Cleavage, day 7, and day 8 blastocysts rates were similar in the three 96-well plates and the control group (*p* > 0.05; Figure 4B). Differences among treatments in blastocyst quality parameters determined by differential apoptotic staining are shown in Table 4. In vitro maturation in flat, ultra-low attachment round-bottom and v-shaped 96-well plates resulted in blastocysts with lower TCN and TE than control (*p* < 0.05). Blastocysts in the R-60 group also presented lower ICM compared with control (*p* = 0.01). However, there were no differences in the ICM/TCN or AC/TCN ratios among treatments and control (*p* > 0.05).

Main results from both culture systems (LM and 96-well plates) are summarized in Table S2.

## 4. Discussion

Currently, most standard oocyte IVM is performed in two-dimensional culture systems, which are both economical and practical. However, these systems have some limitations such as cell flattening and/or decreased interaction among cells that might affect their developmental potential. Therefore, novel alternative systems to mature oocytes might enhance the interaction between the oocyte, the cumulus cells, and different factors within the culture medium, and consequently improve the developmental competence of the oocyte. In this study, we used liquid marbles as microreactors to perform serum-free IVM for the first time in the bovine model. Moreover, we also tested a simple and practical system using differently shaped and coated 96-well plates. We found that both liquid marbles and 96-well plates had a similar effect as 2D-control culture systems in terms of oocyte nuclear maturation, while embryo development was similar after oocyte maturation in the 96-well plates but lower in LM.

We proved that meiotic resumption is not affected in bovine oocytes by the use of LM, since the proportion of oocytes that reached metaphase II was similar in both 2D-controls and liquid marbles systems, which concord with previous studies in cats [41] and sheep [33,34]. In our study, although oocytes matured in LM were able to reach the blastocyst stage, the blastocyst yield was reduced compared with oocytes matured in 2D-controls. Moreover, embryos derived from oocytes matured in LM exhibited a lower total cell number count than those matured in a standard 2D system. It has been shown that oocyte/medium ratios ranging between 1:1 and 1:10 during in vitro maturation do not influence embryo development in cattle [46], hence the slightly different ratio between the control group (1:8) and the 2D droplets and LM (1:6) provides no explanation for the lower total cell number observed in these last two groups. On the other hand, it has been demonstrated that material toxicity can decrease the total cell number and affect embryo rates [47,48]. Still, the toxicity of treated fumed silica particles has not been tested in oocytes. Therefore, we hypothesize that treated fumed silica particles could exhibit some toxicity in bovine oocytes since our findings differ from the results obtained by Bebbere et al., who matured ovine oocytes in LM formed with the same particles, showing only a tendency of the LM to improve the blastocyst rate compared with those matured in 2D conditions [34]. In a previous study of the same group, a similar blastocyst yield was obtained after maturation of ovine oocytes in polytetrafluoroethylene marbles compared with group culture in 2D [33]. On the other hand, in pig oocytes, although embryo development has not been assessed, Gorczyca et al. (2020) [24] performed ultrastructural evaluation of COCs after IVM in fluorinated ethylene propylene marbles, and interestingly, liquid marbles were able to maintain the 3D organization by preventing their flattening and consequent disruption of gap junctions. It is noteworthy that both ovine and porcine results were obtained in serum-containing maturation medium. Although our blastocyst rates in a serum-free system are within the range of serum-containing bovine in vitro embryo culture systems (20–40% [49]), serum-free systems are more prone to toxic influences [50,51,52] and the absence of serum during maturation might be more relevant in the LM system. Moreover, additional manipulation during the LM preparation compared with the standard system may also have affected the developmental capacity of the oocytes. 

Besides the LM system, in the current study, we evaluated the effect of different geometry surfaces on oocyte developmental competence. Although we tested a range of medium volumes and evidenced the importance of paraffin oil overlay when low volumes are used, we did not find differences in oocyte nuclear maturation, embryo development, or embryo quality among the three surface geometries tested. Despite the fact that blastocysts derived from oocytes matured in 96-well plates presented similar ICM/TCM and AC/TCN ratios than 2D-controls, the TCN count was lower than those derived from oocytes matured in standard conditions. These results may be due to certain toxicity, resulting in an impact on embryo quality. However, trophectoderm growth in in vitro embryos was clearly not adversely affected by culture conditions and this fact might give the false impression of good-quality blastocyst production when solely morphological criteria or total cell numbers are used to evaluate embryo quality [53]. The only previous study we found that compared flat, round bottom and v-shaped 96-well plates was not performed on embryos but on cell lines [43]. In this study, different human cell lines, namely A549 (alveolar epithelial), ARPE-19 (retinal epithelial), and Malme-3M (dermal fibroblast), were used, of which the cells attached and spread differently in each plate, but the phenotype and functionality of the cells were not affected by the surface topography [43]. Apart from the different surface geometries, in our study, we investigated both non-treated and ultra-low attachment plates; here, the latter are used to prevent protein adsorption to the culture surface, helping to minimize monolayer cell adhesion to the culture vessel. Therefore, initially, we hypothesized that ultra-low attachment 96-well plates might limit COCs flattening and, consequently, improve developmental rates. However, although ultra-low attachment plates facilitated handling of COCs, we did not find any effect on the evaluated outcomes compared with the non-treated plates.

In order to implement new systems, it is necessary to first study their impact on fundamental cellular processes. In in vitro embryo production, oocyte maturation and embryo development and quality are the main outcome parameters to evaluate culture systems. In our study, we showed that oocyte meiotic resumption was not affected by any of the culture systems. However, only nuclear maturation was assessed and further studies to evaluate differences in cytoplasmic maturation may be performed. On the other hand, despite finding inferior blastocyst quality in both systems and a lower blastocyst rate in LM, we were unable to identify the reason for these declines. Therefore, additional toxicity testing or molecular analyses may further explain our findings. Besides, we thought that our systems would help to preserve the cells’ three-dimensional structure, which would have been translated into an increase in our outcome parameters, thus no ultrastructural evaluation was performed. Furthermore, although LM, v-shaped, and round-bottom 96-well plates enhance the convergence of cumulus-oocyte complexes and secreted factors on the bottom of the marble or well, consequently improving cell interaction (Figure S1); it must be acknowledged that they are still static systems. 

Interestingly, our experiments with different surface topographies exhibited a similar blastocyst rate in comparison with traditional culture but LM reduced it. Yet, alternative culture systems entail higher costs and complexity than conventional IVM (Table 5). Liquid marbles demonstrated the highest level of complexity and risk of loss of oocytes during the elaboration of the marble, with the longest handling time, which might be an additional explanation for the lower blastocyst yield. On the other hand, 96-well dishes made the handling of COCs during preparation and recovery more difficult due to the smaller diameter of the wells. 

## 5. Conclusions

For the first time, liquid marbles and shaped 96-well plates were tested in bovine IVM. We showed that there are differences in development and quality in embryos resulting from oocytes cultured in the 2D control system compared with 3D LM and 96-well plate systems. Importantly, no adverse results were observed in terms of embryo development when cultured in 96-well plates. The decrease that we observed in embryo yield after IVM in LM stresses the importance of using serum-free culture media, as we did, when testing novel recipients for bovine oocyte and embryo culture in order to control for possible toxic effects.

## Figures and Tables

**Table 1:** Nuclear maturation assessment of oocytes matured in: (A) control group, (B) 2D droplets, and (C) liquid marbles (LM).

Treatment	No. Oocytes	GV	GVBD	MI	MII	Degenerated
Control Group	69	0 ± 0	0 ± 0	13 ± 6.9	87 ± 4.1	0 ± 0
2D Droplets	53	0 ± 0	0 ± 0	13.2 ± 6.4	84.9 ± 4.9	1.8 ± 0.2
LM	60	0 ± 0	5 ± 2.8	3.3 ± 0.8	88.3 ± 4.1	3.3 ± 0.2

Germinal vesicle (GV), germinal vesicle breakdown (GVBD), metaphase I (MI), metaphase II (MII), and degenerated ratio of oocytes analyzed with Hoechst staining. Results are expressed as least square means ± standard error (LSM ± SE). No statistical differences were found between groups.

**Table 2:** Effect of the oocyte in vitro maturation in LM on embryo quality.

Treatment	No. Blastocyst	Cell Numbers				ICM/TCN Ratio	AC/TCN Ratio
		TCN	ICM	TE	AC		
Control Group	52	117.8 ± 8.6 ^a^	36.1 ± 2.2 ^a^	81.6 ± 4.0 ^a^	1.9 ± 3.7	30.9 ± 1.3	2.0 ± 0.5 ^a^
2D Droplets	51	82.3 ± 5.1 ^b^	26.2 ± 2.2 ^b^	56.3 ± 4.0 ^b^	2.8 ± 3.7	32.6 ± 1.4	3.9 ± 0.5 ^b^
LM	53	91.3 ± 5.1 ^b^	31.6 ± 2.2 ^ab^	59.8 ± 4.0 ^b^	3.0 ± 3.6	33.7 ± 1.3	3.5 ± 0.5 ^ab^

Total cell number (TCN), trophectoderm cells (TE), inner cell mass (ICM), apoptotic cells (AC), ICM/TCN ratio, and AC/TCN ratio of day-8 blastocyst differentially stained. Different superscripts per column (a and b) represent statistical differences (*p* < 0.05) among groups. Results are expressed as least square means ± standard error (LSM ± SE).

**Table 3:** Nuclear maturation assessment of oocytes matured in: (A) control group, (B) F-60, (C) V-60, and (D) R-60.

Treatment	No. Oocytes	GV	GVBD	MI	MII	Degenerated
Control Group	38	2.6 ± 0	7.9 ± 4.4	2.6 ± 0.1	86.8 ± 5.5	0 ± 0
F60	53	3.8 ± 0	11.3 ± 4.4	0 ± 0	84.9 ± 4.9	0 ± 0
V60	51	0 ± 0	5.9 ± 3.3	3.9 ± 0.1	90.2 ± 4.2	0 ± 0
R60	45	4.4 ± 0	4.4 ± 3.1	0 ± 0	91.1 ± 4.2	0 ± 0

Germinal vesicle (GV), germinal vesicle breakdown (GVBD), metaphase I (MI), metaphase II (MII), and degenerated ratio of oocytes analyzed with Hoechst staining. Results are expressed as least square means ± standard error (LSM ± SE).

**Table 4:** Effect of the oocyte in vitro maturation in three different surface geometries on embryo quality.

Treatment	No. Blastocyst	Cell Numbers				ICM/TCN Ratio	AC/TCN Ratio
		TCN	ICM	TE	AC		
Control Group	80	134.6 ± 5.2 ^a^	51.0 ± 2.5 ^a^	83.6 ± 3.6 ^a^	1.8 ± 2.6	38.6 ± 3.9	1.7 ± 0.3
F60	83	112.4 ± 5.1 ^b^	44.8 ± 2.4 ^ab^	67.5 ± 3.5 ^b^	2.0 ± 2.5	34.5 ± 3.9	2.2 ± 0.3
V60	84	112.2 ± 5.1 ^b^	44.1 ± 2.4 ^ab^	68.0 ± 3.5 ^b^	1.7 ± 2.5	39.4 ± 3.8	1.7 ± 0.3
R60	70	97.6 ± 5.6 ^b^	39.8 ± 2.7 ^b^	57.8 ± 3.8 ^b^	1.61 ± 2.5	41.9 ± 4.2	1.7 ± 0.3

Total cell number (TCN), trophectoderm cells (TE), inner cell mass (ICM), apoptotic cells (AC), ICM/TCN ratio, and AC/TCN ratio of day-8 blastocyst differentially stained. Different superscripts per column (a and b) represent statistical differences (*p* < 0.05) among groups. Results are expressed as least square means ± standard error.

**Table 5:** Characteristics of in vitro maturation systems.

Maturation Technique	Description	Hands-on-Time (min)		Complexity	Cost (€)
		Preparation	Recovery		
Four well dish without oil (control)	Sixty COCs in 500 μL of maturation medium in flat-bottom 4-well dishes without oil covering	1	0.5	+	1.3
Droplets on Petri dish under oil (2-D Droplets)	Five COCs in droplets of 30 μL of maturation medium in a Petri dish (60 × 15 mm) and covered with 7.5 mL paraffin oil	4	3	++	3.2
Liquid marbles	Five COCs in 30 μL of maturation medium are placed on top of a layer of treated fumed silica powder to form a LM, which is transferred to a well of a 24-well plate with a cut 1000 μL pipette tip	15	7	++++	3.3
96-well plates differently shaped	Five COCs in 60 μL of maturation medium covered by 30 μL of paraffin oil overlay in flat, ultra-low attachment round-bottom, and v-shaped 96-well plates	4	5	+++	Flat: 3.8 V-shaped: 3.9Round-bottom: 22.3

Comparison of in vitro maturation systems in terms of working principle, hands-on-time (i.e., based on the manipulation time in minutes required to handle 60 COCs from the dish in which they are selected and washed in HEPES-TALP to the final in vitro maturation (IVM) system (preparation) and from the IVM system to the fertilization dish (recovery)), ease-of-use and approximate cost for one maturation process (i.e. based on cost of the dish, oil if necessary and silica powder for liquid marbles preparation). + = low; ++ = moderate; +++ = high; ++++ = very high.

## Data Availability

All data generated or analyzed during this study were included in the manuscript and its Supplementary Materials. Raw data are available from the corresponding author upon reasonable request.

## Supplementary Materials

The following supporting information can be downloaded at: https://www.mdpi.com/article/10.3390/ani13101635/s1, Table S1: Developmental results of the pilot study in v-shaped 96-well plates; Figure S1: Organization of oocytes in three differentially shaped 96-well plates; Table S2: Summary of main results of the tested systems; Figure S2. Representative images of bovine day 8 blastocysts; Figure S3: Differential apoptotic staining of day 8 blastocyst.
