# Supplementary material for: Alternative Culture Systems for Bovine Oocyte In Vitro Maturation: Liquid Marbles and Differentially Shaped 96-Well Plates
Source: Animals (Basel). 2023 May 14;13(10):1635. doi: 10.3390/ani13101635 (PMC10215113; doi:10.3390/ani13101635)
Supplement: Supplementary file 1 [file animals-13-01635-s001.zip › animals-2368006-supplementary.pdf]

Supporting information

Table S1. Developmental results of the pilot study in v-shaped 96-well plates.

Treatment	No. embryos	Cleavage	Blastocyst Day 7	Blastocyst Day 8
Control Group	252	78.6 ± 2.6	19.8 ± 2.5	31.7 ± 2.9
V-60	155	84.5 ± 2.9*	21.3 ± 3.3	34.2 ± 3.8
V-120	148	79.1 ± 3.3	20.9 ± 3.3	29.1 ± 3.7
OV-30	160	73.1 ± 3.5	17.5 ± 3.0	28.1 ± 3.6
OV-60	163	79.1 ± 3.2	21.5 ± 3.2	35.6 ± 3.8
OV-120	143	69.9 ± 3.8*	22.4 ± 3.5	34.3 ± 4.0

Cleavage, day 7 and day 8 blastocyst rates expressed as a percentage of presumed zygotes. * Significant differences ($P < 0.05$). Results are expressed as least square means ± standard error.

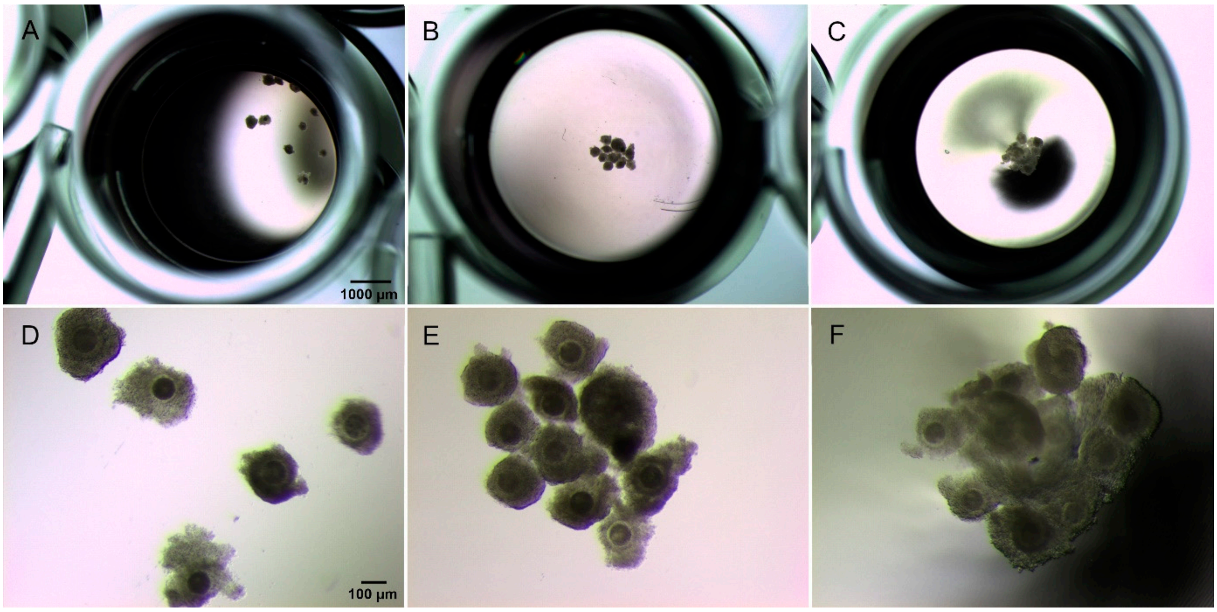

Figure S1. Organization of oocytes in three differentially shaped 96-well plates. The images show differences in interactions between immature cumulus-oocyte complexes when cultured in three surface geometries: flat (A, D), ultra-low attachment round-bottom (B, E), and v-shaped (C, F) 96-well plates.

Table S2. Summary of main results of the tested systems.

	Treatment	MII (%)	Blastocyst Day 8 (%)	TCN	ICM/TCN	AC/TCN
Exp. 1	Control Group	87 ± 4.1	40.1 ± 4.6	117.8 ± 8.6^{a}	30.9 ± 1.3	2.0 ± 0.5^{a}
	2D Droplets	84.9 ± 4.9	38.8 ± 4.2	82.3 ± 5.1^{b}	32.6 ± 1.4	3.9 ± 0.5^{b}
	LM	88.3 ± 4.1	26.1 ± 3.7	91.3 ± 5.1^{b}	33.7 ± 1.3	3.5 ± 0.5^{ab}
Exp. 2	Control Group	86.8 ± 5.5	36.4 ± 2.4	134.6 ± 5.2a	38.6 ± 3.9	1.7 ± 0.3
	F-60	84.9 ± 4.9	41.3 ± 2.8	112.4 ± 5.1b	34.5 ± 3.9	2.2 ± 0.3
	V-60	90.2 ± 4.2	37.3 ± 2.5	112.2 ± 5.1b	39.4 ± 3.8	1.7 ± 0.3
	R-60	91.1 ± 4.2	38.8 ± 2.5	97.6 ± 5.6b	41.9 ± 4.2	1.7 ± 0.3

Metaphase II (MII) ratio of oocytes analyzed with Hoechst staining. Day 8 blastocyst rates expressed as a percentage of presumed zygotes. Different superscripts per column (a and b) represent statistical differences ($P < 0.05$) among groups. Results are expressed as least square means ± standard error.

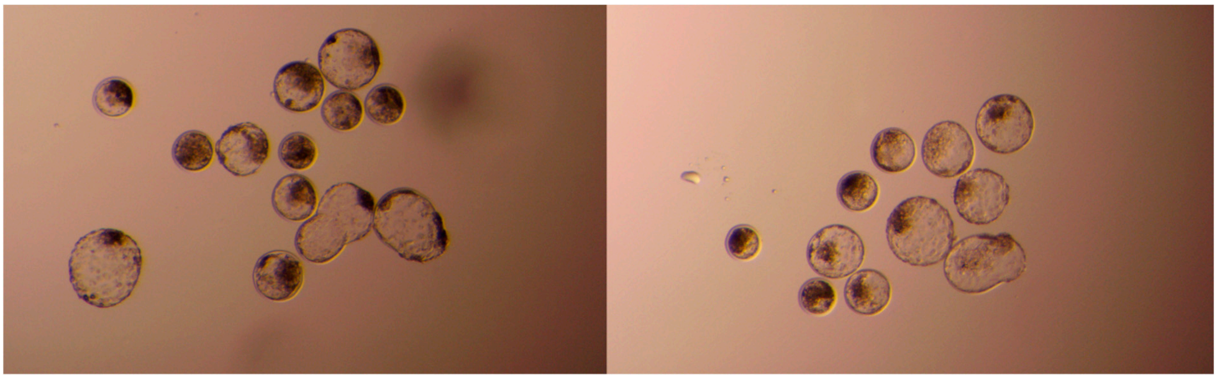

Figure S2. Representative images of bovine day 8 blastocysts.

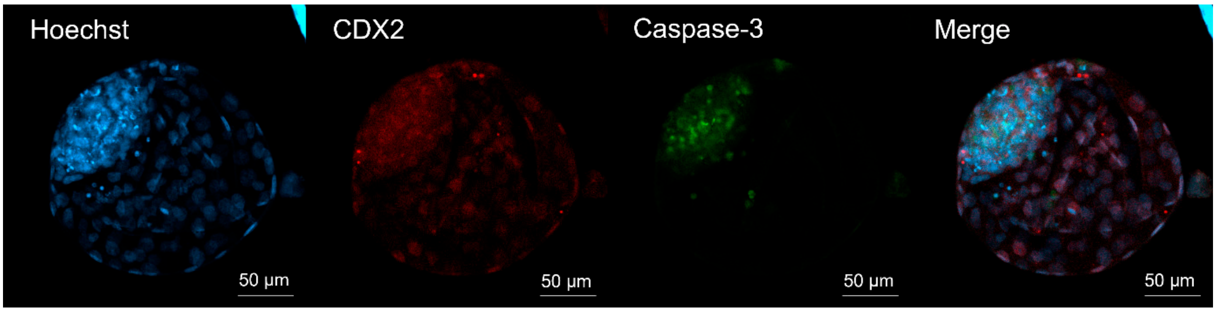

Figure S3. Differential apoptotic staining of day 8 blastocyst. Hoechst 33342 (blue fluorescence) was used to stain the nuclei of both trophectoderm and inner cell mass cells; CDX2 (red fluorescence) was used to specifically stain trophectoderm cells; and anti-caspase 3 (green fluorescence) was used to stain apoptotic cells.
